# Supplementary figures and images for: Zn2+ is essential for Ca2+ oscillations in mouse eggs
Source: eLife. 2023 Dec 15;12:RP88082. doi: 10.7554/eLife.88082 (PMC10723796; doi:10.7554/eLife.88082)

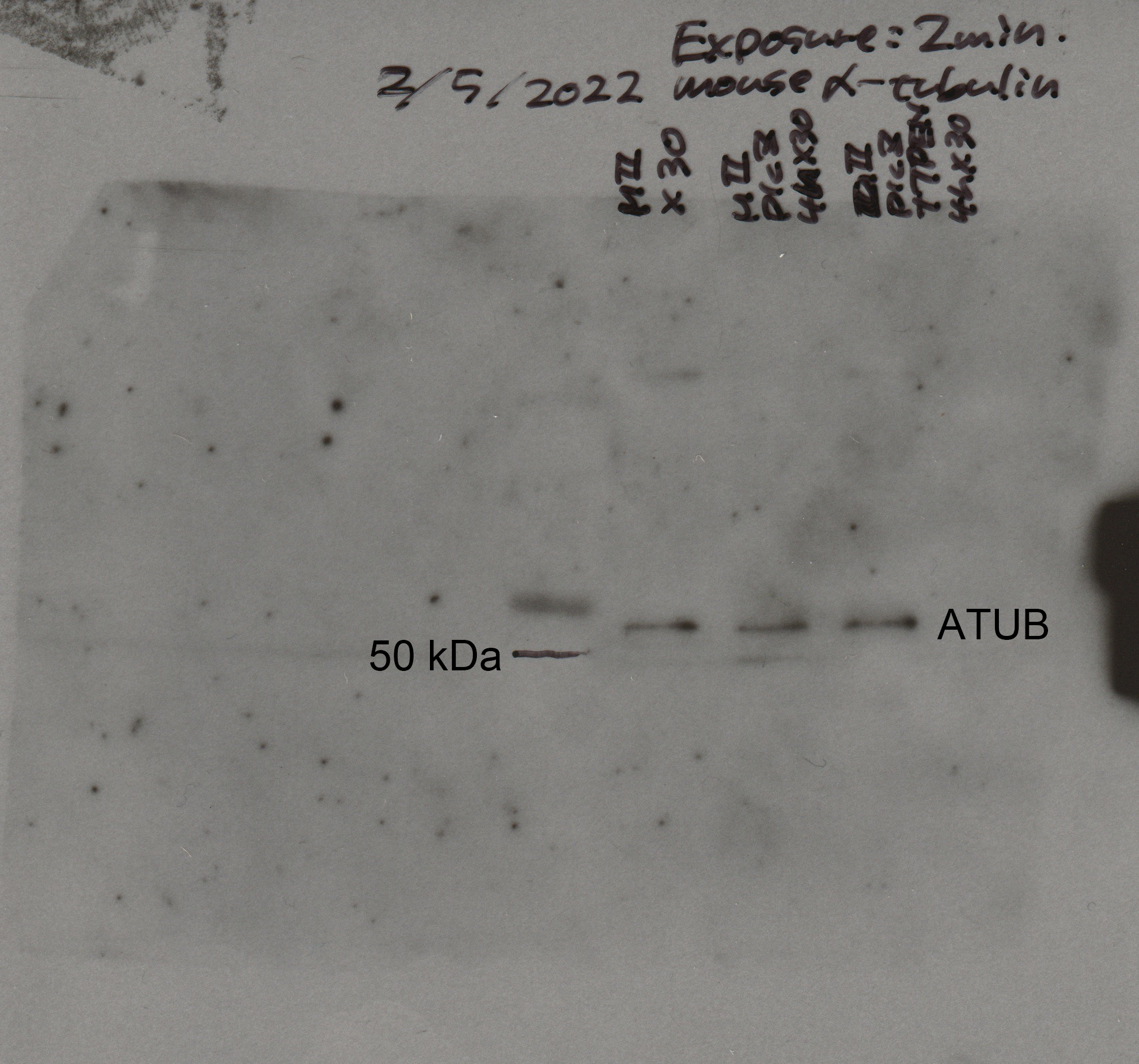

Supplement: Figure 2—source data 1. [file elife-88082-fig2-data1.zip › Figure 2-source data 1/Figure 2F-blot-ATUB-labeled.tif]

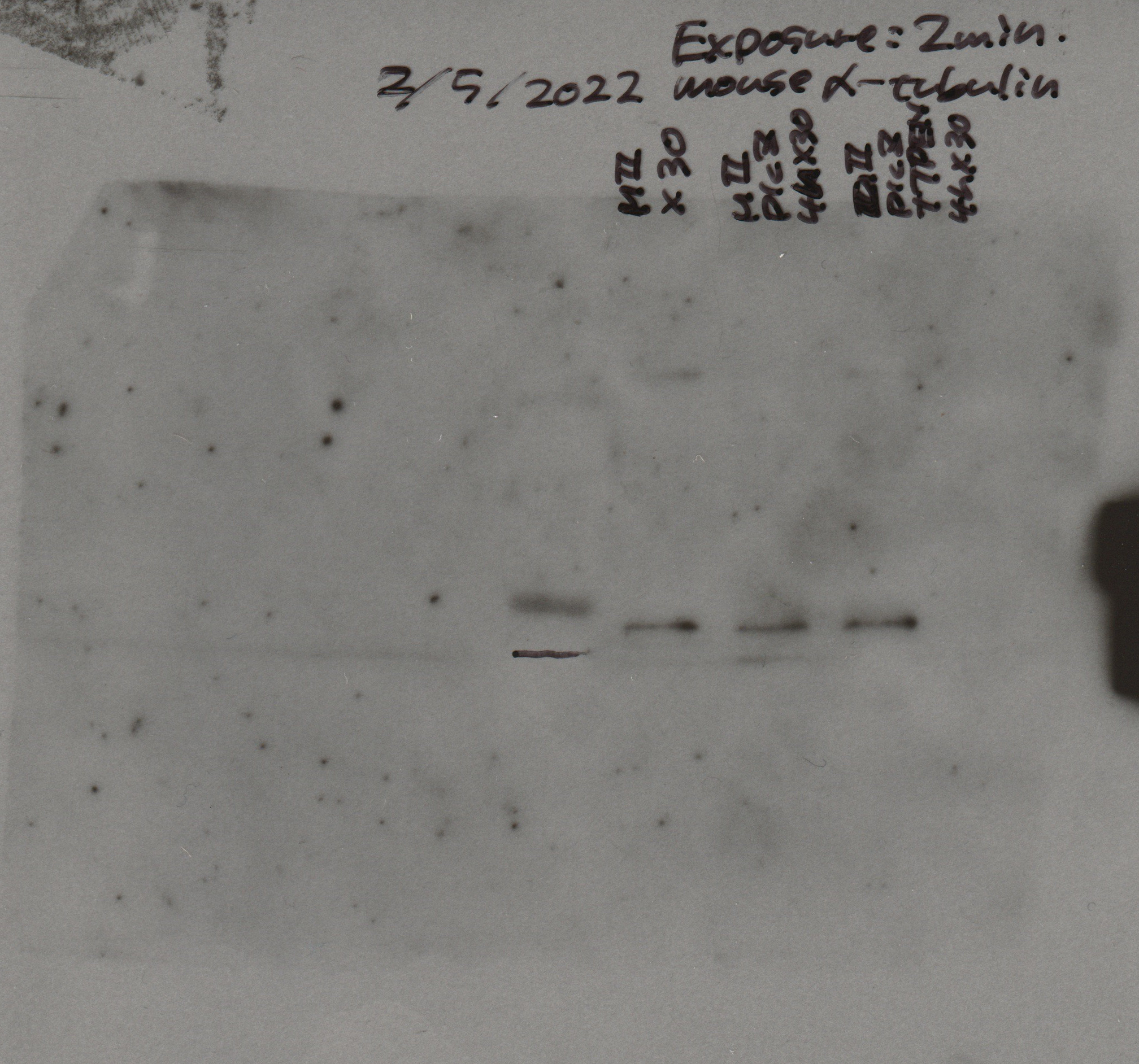

Supplement: Figure 2—source data 1. [file elife-88082-fig2-data1.zip › Figure 2-source data 1/Figure 2F-blot-ATUB-unlabeled.tif]

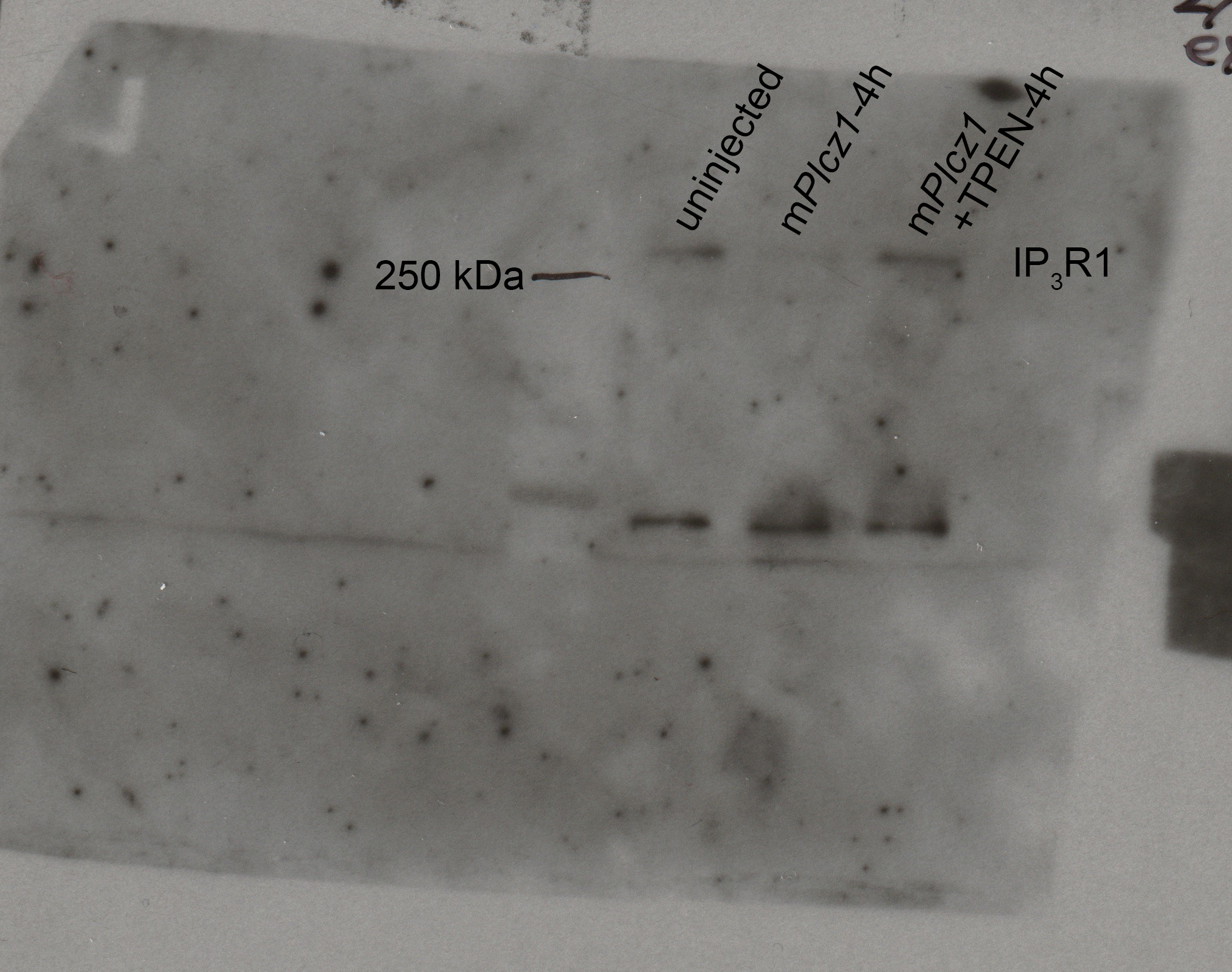

Supplement: Figure 2—source data 1. [file elife-88082-fig2-data1.zip › Figure 2-source data 1/Figure 2F-blot-IP3R1-labeled.tif]

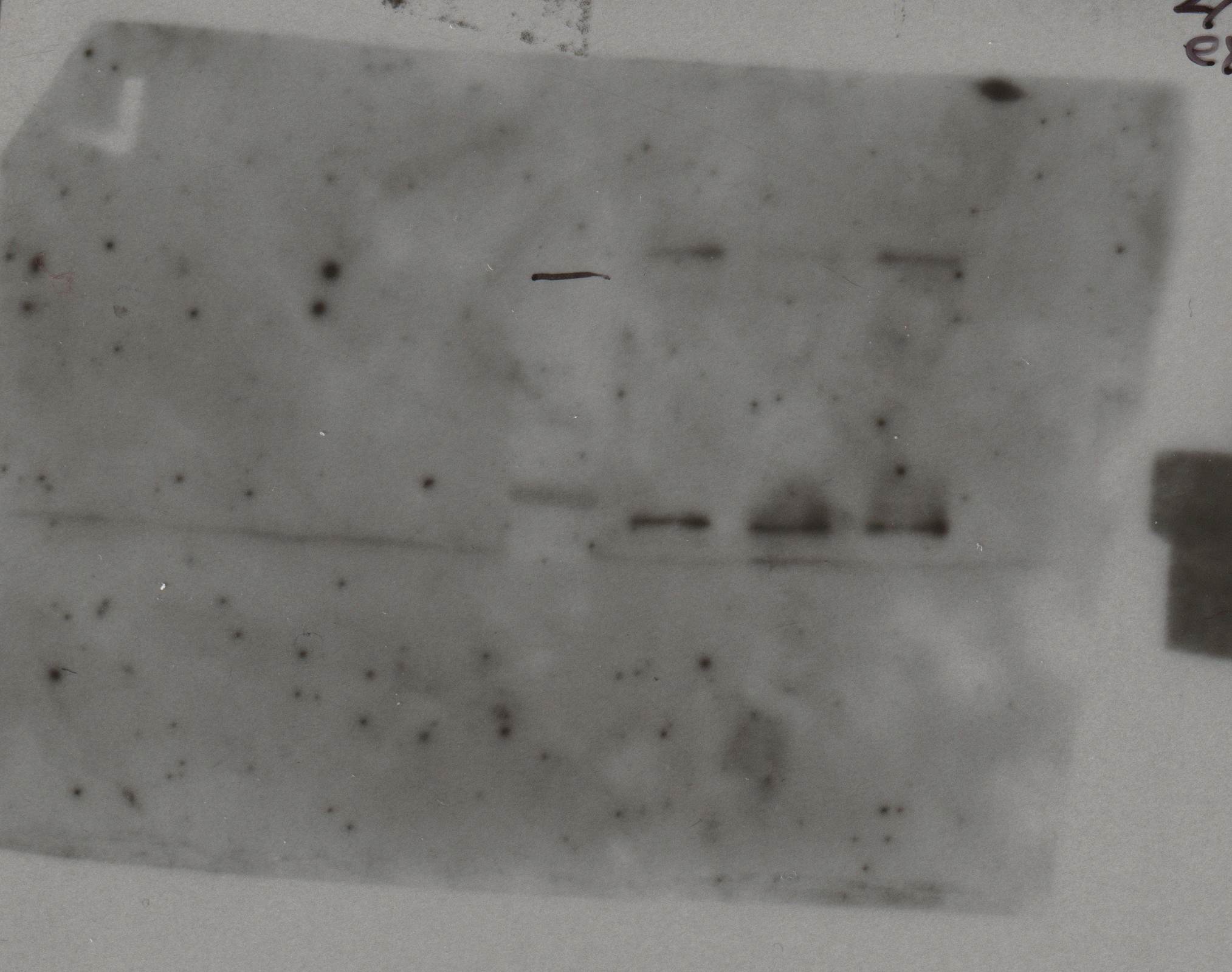

Supplement: Figure 2—source data 1. [file elife-88082-fig2-data1.zip › Figure 2-source data 1/Figure 2F-blot-IP3R1-unlabeled.tiff]

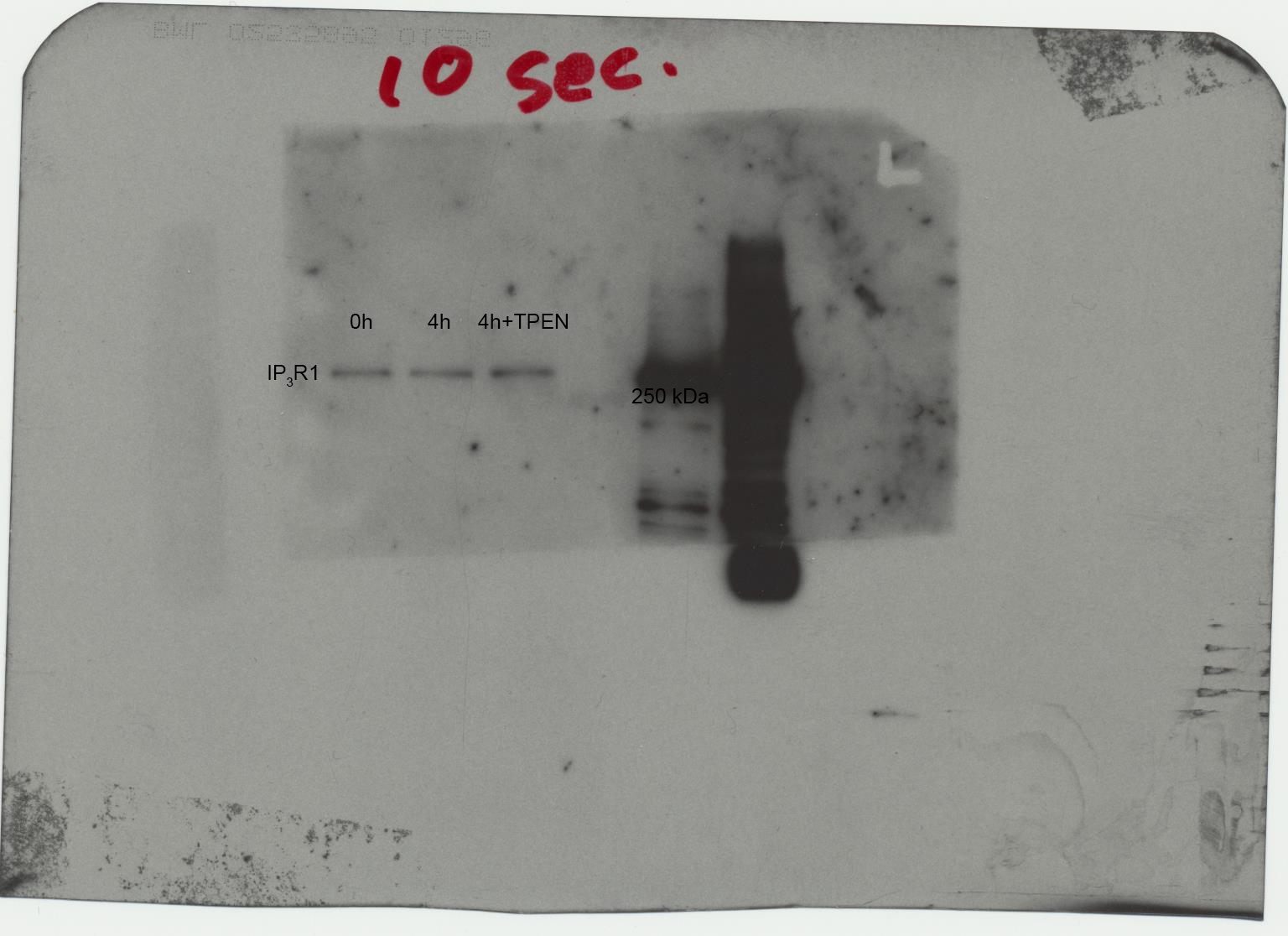

Supplement: Figure 4—source data 1. [file elife-88082-fig4-data1.zip › Figure 4-source data 1/Figure 4C-blot-IP3R1-labeled.tif]

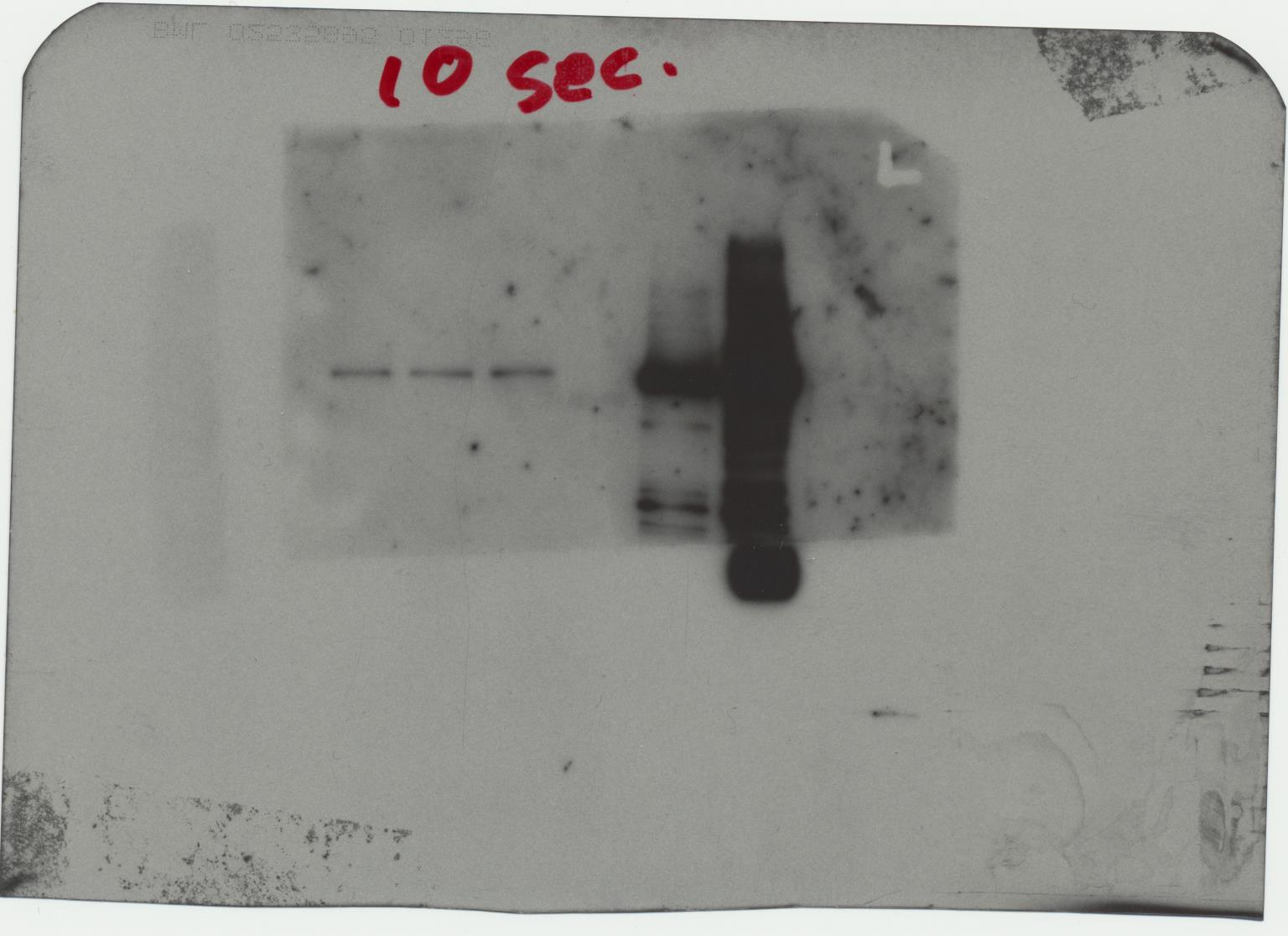

Supplement: Figure 4—source data 1. [file elife-88082-fig4-data1.zip › Figure 4-source data 1/Figure 4C-blot-IP3R1-unlabeled.tiff]
